# The evolution of molecular hydrogen: a noteworthy potential therapy with clinical significance

**DOI:** 10.1186/2045-9912-3-10

**Published:** 2013-05-16

**Authors:** Brandon J Dixon, Jiping Tang, John H Zhang

**Affiliations:** 1 Department of Physiology, Loma Linda University School of Medicine, Risley Hall, Room 223, Loma Linda, CA, 92354, USA; 2 Department of Neurosurgery, Loma Linda University School of Medicine, Loma Linda, CA, USA

**Keywords:** Antioxidant, Cytoprotection, Hydrogen therapy, Mechanisms, Reactive oxygen species

## Abstract

Studies on molecular hydrogen have evolved tremendously from its humble beginnings and have continued to change throughout the years. Hydrogen is extremely unique since it has the capability to act at the cellular level. Hydrogen is qualified to cross the blood brain barrier, to enter the mitochondria, and even has the ability to translocate to the nucleus under certain conditions. Once in these ideal locations of the cell, previous studies have shown that hydrogen exerts antioxidant, anti-apoptotic, anti-inflammatory, and cytoprotective properties that are beneficial to the cell. Hydrogen is most commonly applied as a gas, water, saline, and can be applied in a variety of other mediums. There are also few side effects involving hydrogen, thus making hydrogen a perfect medical gas candidate for the convention of novel therapeutic strategies against cardiovascular, cerebrovascular, cancer, metabolic, and respiratory diseases and disorders. Although hydrogen appears to be faultless at times, there still are several deficiencies or snares that need to be investigated by future studies. This review article seeks to delve and comprehensively analyze the research and experiments that alludes to molecular hydrogen being a novel therapeutic treatment that medicine desperately needs.

## Introduction

### History

**Figure 1:**
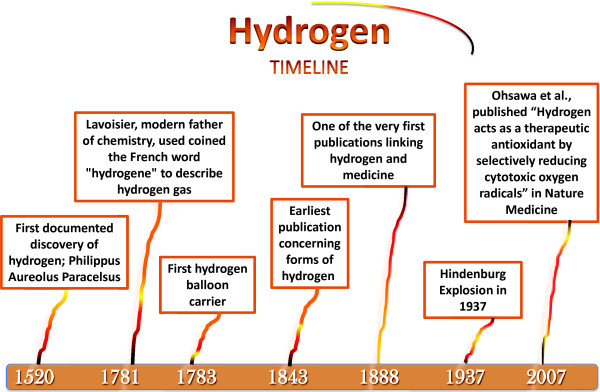
**The progress of hydrogen in history.** A timeline of the history of molecular hydrogen [1-3].

Hydrogen has been shown to be an extremely useful element that has been used in a vast range of disciplines. Since its initial discovery, hydrogen has been effectively applied in a variety of combinations with other elements and different physical states. The role of hydrogen is constantly evolving from its humble beginnings in the chemistry field as a mysterious flammable gas, to its aeronautic applications in balloons, and its emerging role as a potential therapy in medicine (See Figure 1).

The first documented discovery of hydrogen was by Philippus Aureolus Paracelsus in 1520. Paracelsus unknowingly discovered a flammable gas by burning some metal with an acid and collecting the products (Royal Chemistry Society). After Paracelsus discovered this mysterious flammable gas, others replicated the process and began working with the gas. However, hydrogen gas never had an official or common name. It was not until 1783, that Lavoisier, who is often referred to as the modern father of chemistry, used the French word “hydrogene” to describe the gas (Royal Chemistry Society).

The first applications of hydrogen were of the aeronautical nature. In 1783, Frenchmen Jacques Charles created the first hydrogen balloon carrier. Since then and throughout time many other forms of hydrogen filled balloons would follow along with some success and disasters. One of the most infamous disasters involving hydrogen gas is the explosion of Hindenburg, a German passenger aircraft utilizing hydrogen gas [4].

### Characteristics

Hydrogen can be characterized as the lightest and most abundant chemical element. A large amount of hydrogen is usually found in water and organic compounds, which causes free hydrogen to be rare on Earth [5]. According to the Hazardous Substances Data Bank, hydrogen is also an odorless, tasteless, colorless gas [6].

As a result of its unique features hydrogen has many advantageous characteristics. One major advantage that hydrogen contains is its ability to diffuse through membranes and enter the cytosol. Hydrogen can also enter the mitochondria and nucleus. This is extremely favorable since many known antioxidants lack the ability to target organelles and are not as effective in this manner. Molecular hydrogen is also believed to be advantageous in medical procedures since it is able to maneuver through the blood brain barrier. There also are few side effects involving hydrogen. It is proposed that few side effects occur, since it seems that hydrogen reacts with strong oxidants and its levels does not seem to interfere with cell signaling processes involving reactive oxygen species [2].

Out of the numerous observations evolved the applications of hydrogen. In 1888, the Annals of Surgery had recorded one of the very publications linking hydrogen and medicine. At that point in time unnecessary laparotomies were often performed since it was very difficult for surgeons to determine visceral injuries to the intestines and the stomach. It was also reported that a surgeon was able to use hydrogen gas to insufflate the gastro-intestinal canal to accurately determine and locate visceral injuries, avoiding unwarranted surgeries [3].

### Modern uses

Today, hydrogen is still very instrumental and can be found in an assortment of fashions concerning medicine and scientific research. One medicinal approach that employs hydrogen is the breath hydrogen test. The breath hydrogen test is performed by measuring the amount of hydrogen that is produced by intestinal bacteria that are constantly synthesizing hydrogen as a result of fermentation of unabsorbed carbohydrates [7]. The analysis of the results of the breath hydrogen test can serve as biomarkers and can be also used to compute oral-cecal transport, transit times, and overgrowth of bacteria. The breath hydrogen test is also used as biomarkers in clinical and scientific research ranging from biochemistry, dentistry, and physiology [5].

In 2007, Ohsawa et al., published “Hydrogen acts as a therapeutic antioxidant by selectively reducing cytotoxic oxygen radicals” in Nature Medicine. Ohsawa et al., reported that hydrogen is able to react with cytotoxic oxygen radicals and protect against oxidative damage. These conclusions were made based upon experiments observing a rat model of focal ischemia and reperfusion. After ischemia was induced and reperfusion performed, it was observed that arterial blood contained elevated levels proportionate to the concentration of hydrogen that was inhaled. Also, it is suggested that the tissue was able to absorb hydrogen, since dissolved hydrogen was found at lower levels than the arterial blood. The study also suggests that hydrogen is able to prevent oxidative damage by reacting with the hydroxyl radical. This is important since the hydroxyl radical is believed to be the most dangerous oxygen species since there are no naturally occurring mechanisms to prevent its affects. As a result of the findings by Ohsawa et al., the convention of hydrogen has yet evolved. This publication has sparked many investigations concerning hydrogen as a selective reactive oxygen species scavenger and it its potential as an antioxidant therapy [2].

## Mechanisms

### Anti-oxidation

The exact mechanisms of how hydrogen acts still remain a mystery in the scientific community. One useful finding by Kayar et al. showed that mammalian tissues under hyperbaric conditions do not oxidize hydrogen, thus leading to hydrogen being used as a non-metabolic portion of breathing gas for deep divers [8]. However more mysteries still remain. Mechanisms of hydrogen serving as an antioxidant scavenger (specifically the hydroxyl radical) have been established and confirmed by a number of scientists. However the exact mechanism of this scavenging ability has not yet been discovered.

In 1975, Dole et al., was among one of the first to propose that hydrogen had antioxidant abilities against alkyl radicals and the hydroxyl radical. Dole et al., made the initial observations and proposed that hydrogen contained anti-cancer properties since hyperbaric hydrogen therapy degenerated squamous cell carcinomas. It was reported that hydrogen therapy also had the capability of scavenging the hydroxyl radical by means of an exothermic reaction. When an exothermic reaction occurs hydrogen combines with the hydroxyl radical to form water and hydrogen. The hydrogen generated from the water forming reaction is then able to combine with the superoxide radical, which causes another reaction and prevents the formation of more peroxide and hydroxyl radicals.

**Figure 2:**
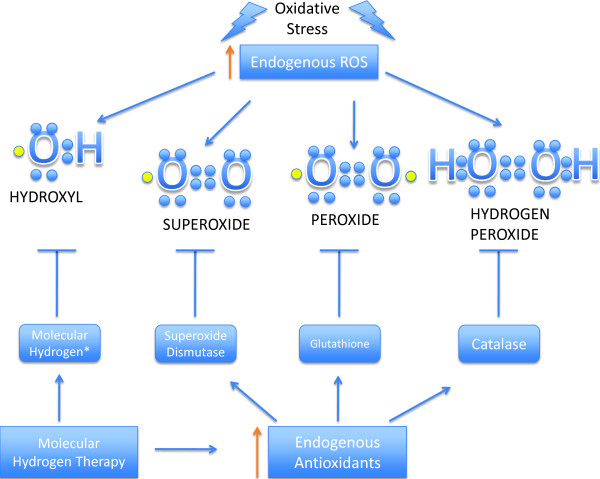
**Hydrogen therapy reduces hydroxyl radical and increases endogenous antioxidants caused by oxidative stress.** Molecular hydrogen is proposed to be protective by increasing endogenous antioxidants in addition to scavenging the hydroxyl radical after an injury such as oxidative stress [9,10].

More recent studies have also shown that hydrogen is able to reduce ROS in vitro, as a result of its proposed ability to scavenge the hydroxyl radical, “the most potent oxidant known to mankind” [9,10]. When free radicals or ROS accumulates, usually as a result of cellular processes, it leads to oxidative stress. Oxidative stress can cause serious damage to tissues and can lead to a variety of diseases. It is also proposed that hydrogen is capable of organ protection in cerebral ischemia, neonatal cerebral hypoxia ischemia, liver injuries, lung injuries, and myocardial injuries all caused by ischemia reperfusion, a condition that causes an increase in oxidative stress. In addition it has been shown that hydrogen is able to increase antioxidant enzymes to help suppress the disastrous effects of oxidative stress. A summary of the antioxidant properties of hydrogen therapy is described in Figure 2[10].

### Anti-inflammation

Hydrogen has also been described as containing anti-inflammatory properties. Gharib et al. observed these anti-inflammatory properties in a mouse modeling chronic liver inflammation induced by the parasite *Schistosoma mansoni*. It was suggested that hyperbaric hydrogen treatment was able to improve liver hemodynamics and reduce portal hypertension, as well as reduce liver fibrosis by attenuating inflammatory cytokines [11].

### Cytoprotection

Although hydrogen has been strongly implicated with reducing oxidative stress, it has been proposed that hydrogen effects signaling mechanisms and can also induce cytoprotective factors [12,13].

In 2011, Itoh et al., demonstrated that hydrogen was able to affect signal transduction and act as a signal modulator. Hydrogen was able to act in this manner by inhibiting LPS/IFNγ-induced nitric oxide production in macrophages, resulting in decreased inflammation in Type I allergies. The exact molecules that hydrogen is binding to and modulating are unknown, but it is possible to narrow down and determine specific sites. It was confirmed that hydrogen was able to modulate signal transduction, which suggests that hydrogen is a signal modulator. Further studies need to be conducted to determine exactly how, why, and under what conditions that hydrogen can be a signal modulator [12]. Another proposed mechanism for hydrogen is its ability to provide cytoprotection by increasing other antioxidant enzymes such as superoxide dismutase and catalase [10]. It is also proposed that hydrogen can bestow cytoprotection by preventing the activation of caspase-3, which through a series of events can reduce apoptosis [13]. Shi et al. has also proposed that molecular hydrogen may be able to affect signal transduction by interacting with metalloproteins, since metal ions can be a possible binding site for hydrogen [14].

### Signal modulation

**Figure 3:**
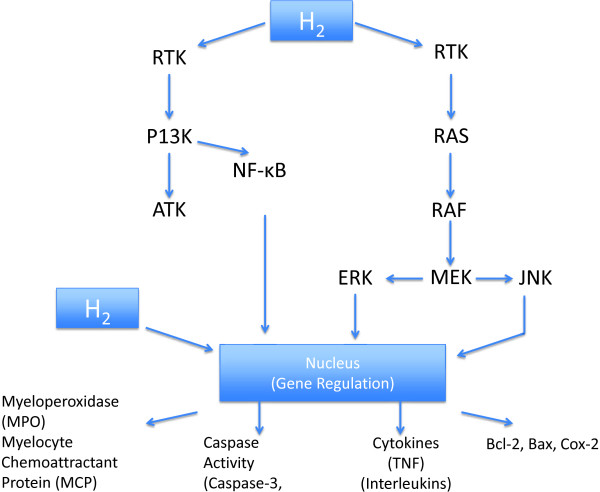
**Possible mechanisms of molecular hydrogen.** Possible pathways for molecular hydrogen. It has been proposed that molecular hydrogen has the capabilities to affect the pathways mentioned and to directly or indirectly assist in the gene regulation or protein expression of the following: MPO, MCP, Caspase-3, Caspase-12, TNF, interleukins, Bcl-2, Bax, Cox-2 [15-20].

Recently, it has been reported that hydrogen may be able to inhibit pathways as a result of its ability to reduce levels of ROS. It has also been shown that molecular hydrogen is able to inhibit the TNF-α/NFκβ pathway as well as the Ras-ERK1/2-MEK1/2 and Akt pathways, these findings as well as possible gene regulatory effects are illustrated in Figure 3[15,16]. The suppression of these pathways by use of hydrogen was demonstrated in neointimal hyperplasia models in rats. Further investigation of the suppression of these pathways should be heavily investigated since these pathways are important; and involved with inflammatory responses, gene regulation, and apoptosis. The possibilities of the effects of regulation of these two pathways and diseases alone are quite high (See Figure 3) [16].

### Administration of hydrogen

Three of the most common ways to administer hydrogen as a treatment are: 1) inhaling hydrogen gas, 2) injecting a hydrogen rich saline, 3) drinking hydrogen rich water. Because hydrogen gas has no foreign smell, it can easily be inhaled into the body [21]. Although some reports have shown that complications involving facemasks and unattended patients, specifically neurologically impaired patients, may cause inconsistencies with inhalation. Generally hydrogen gas can be easily inhaled through facemasks, ventilators, gas chambers, or nasal cannulas [4,22]. It is also possible to administer hydrogen intravenously, by injecting hydrogen rich saline. Hydrogen rich saline can be created by dissolving hydrogen in physiological saline under high pressures. Hydrogen rich saline is also able to provide more accurate concentrations of hydrogen into the body [13]. Hydrogen rich water can be easily generated by exposing water to magnesium by dissolving electrolyzed hydrogen into water, or by dissolving molecular hydrogen into water under high pressures.

Once hydrogen has been generated in its chosen medium, the levels of concentrations can easily be detected as well. The concentration of hydrogen in aqueous and gas solutions can be detected by using electrochemical gas sensors. An alternative detection method that is less expensive is using a methylene blue-platinum colloid reagent [23].

There are also other forms of molecular hydrogen administration, which include topical agents and room air administration [24].

### Potential of hydrogen in the top ten causes of death

**Figure 4:**
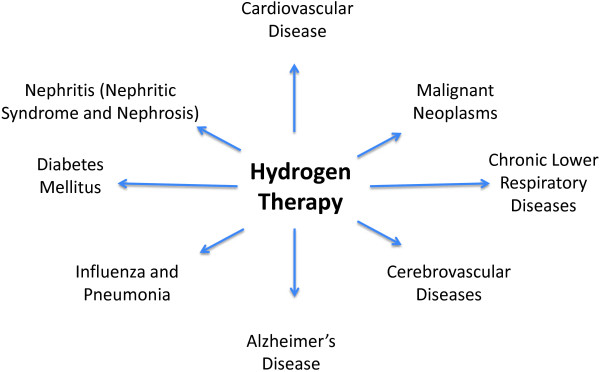
**Hydrogen therapy potential for top causes of death in the US.** The potential for molecular hydrogen in the top causes of death in the United States (excluding deaths caused by suicide and accidents) [23].

At the time of the creation of this document, the most recent data available to the public concerning the top causes of death in the United States of America can be found in a preliminary report by the Center for Disease Control [25]. The CDC describes the following as the top ten causes of death in America: cardiovascular disease, malignant neoplasms, chronic lower respiratory diseases, cerebrovascular diseases, accidents (unintentional injuries), Alzheimer’s disease, diabetes mellitus, influenza and pneumonia, nephritis (nephritic syndrome and nephrosis), and suicide (intentional harm). Interestingly enough Figure 4 shows how hydrogen therapy can either be linked or speculated to be a potential treatment in each of those top causes of death excluding deaths begetting accidents (unintentional injuries) and suicides (intentional harm) (See Figure 4).

### Nephritis and nephritic syndrome

The effects of hydrogen rich water on gentamicin induced nephrotoxicity was analyzed by Matsushita et al. It was discovered that hydrogen rich water was able to improve renal dysfunction caused by nephrotoxicity by reducing oxidative stress as well as lowering serum creatinine (Cr) and blood urea nitrogen (BUN) when compared to control rats [26]. Positive results of hydrogen rich saline were also observed when used as a treatment after renal ischemia and reperfusion injury. Wang et al. observed statistically significant decreases or reversals in MDA levels, blood urea nitrogen levels, creatinine levels, myeloperoxidase (MPO) levels, and pro-inflammatory cytokines (TNF-α, IL-1β, and IL-6) when compared to control counterparts. While increases in the antioxidants superoxide dismutase and catalase as well as reversals in apoptotic death were also observed. These results suggest that hydrogen rich saline is effective in combating renal ischemia and reperfusion injury [17].

One clinical trial that shows promise involves dissolved hydrogen being used as a treatment for haemodialysis patients. To observe the effects of hydrogen on the patients, a dialysis solution containing a high concentration of dissolved hydrogen gas was created and was administered to 21 haemodialysis patients three times a week for a period of six months. During the six months, the blood pressure, skin temperatures, markers for oxidative stress, myeloperoxidase, myelocyte chemoattractant protein (MCP), highly sensitive C-reactive protein, and N terminal pro-brain natriuretic peptide for the patients were all measured and monitored. The results showed that after treatment with hydrogen, high blood pressure generally decreased for the patients and some instances patients reached normotensive statuses. There were also decreases in MCP and MPO, which are chemokines and enzymes secreted by monocytes and neutrophils respectively. The decrease in MCP and MPO is believed to be representative of suppressed inflammatory and white blood cell (specifically neutrophil) responses. The studies concluded that hydrogen was able to quell inflammation and improve blood pressure [27].

The ROS scavenging abilities of hydrogen rich water were demonstrated in experiments conducted by Kitamura et al. Based on the results, the conclusion that hydrogen rich water administration against cisplatin-induced renal nephrotoxicity ameliorated the effects and improved renal dysfunction was made. Altogether hydrogen rich mediums seem to improve the damaging or even reverse in some instances the damaging effects stemming from renal dysfunction and nephrotoxicity; and may have a potential in combating nephritis and nephritic syndrome [28].

### Diabetes mellitus

Skin lesions are a common condition that can develop as a result of diabetes mellitus. Recently, it has been shown that the production of oxidative stress by high levels of blood glucose causes an overproduction of ROS, which may result in a possible pathogenesis to diabetic skin lesions. In a study by Yu et al., hydrogen was applied as a treatment to human skin fibroblast exposed to oxidative stress induced by high levels of glucose and mannose. The results showed that hydrogen was able to improve viability of the cells by exhibiting its antioxidant properties and reducing oxidative products exposed to high glucose and mannose. A loss in membrane potential was exhibited by cells exposed to high glucose and mannose, when compared to the hydrogen treatment group it was observed that hydrogen was able to reduce the loss of membrane potential. The study concluded that overall, hydrogen might be able to play a role in managing oxidative damage in skin lesions induced by diabetes mellitus [29].

Another study involving hydrogen and diabetes mellitus investigated the effects of hydrogen rich water in vitro and in vivo. In the in vitro study, hydrogen rich water was given as a treatment for ROS induced by α,β-dicarbonyl compounds and glucose, which is common to patients with type-2 diabetes. In the in vivo study hydrogen rich water was a treatment for SHR. Cg-Leprcp/NDmcr rats, an animal model for metabolic syndrome. The results showed that hydrogen rich water was in both in vitro and in vivo studies. When in vitro, hydrogen was able to reduce ROS, when analyzed in vivo, hydrogen was also able to reduce renal ROS too. These findings indicate that hydrogen rich water may be a prospective treatment for renal dysfunction in type-2 diabetes mellitus patients [30].

One study using streptozotocin-induced diabetic rats, analyzed the affects of hydrogen rich saline on erectile dysfunction. Erectile dysfunction is more widespread in men with diabetes mellitus than men without the disease. The data shows that there was increased expression of endothelial nitric oxide synthase and increased nitrite and nitrate levels in the corpus cavernosum for the treatment group. Indicating that hydrogen might have restored nitric oxide vasodilation and erectile function via endothelial nitric oxide synthase. Treatment with hydrogen rich saline also revealed that hydrogen restored expression of the anti-apoptotic factor, bcl-2, and decreased protein expression of bax, a pro-apoptotic factor, in the corpus cavernosum, when compared to the controls and diabetic groups. Altogether the results suggest hydrogen rich saline may be an effective therapy to erectile dysfunction in humans as well [18].

Another study involving hydrogen and diabetes mellitus observed the effects of hydrogen rich saline on a diabetic rat model as well as an insulin resistant rat model. The results of the study seem to suggest that hydrogen rich saline may have had an anti-lipidemic effect. Hydrogen rich saline as a treatment for both the diabetic rat model and the insulin resistant rat model may have acted like an anti-lipidemic agent, since the levels of total cholesterol, triglyceride, and low-density lipoproteins were all significantly lowered. These findings are significant because it implies that hydrogen rich saline may be able to play a therapeutic role in insulin resistance and diabetes mellitus [31].

Based upon the knowledge that hydrogen has the capability to reduce oxidative stress, several clinical trials have been performed. Since there are few clinical trials, studies issue warnings about the interpretation of the data found. In order for hydrogen to advance, more clinical trials are needed to fully ascertain the effectiveness of the therapy [32].

In a clinical trial performed by Kajiyama et al., the effects of hydrogen rich water on patients with type 2 diabetes mellitus and impaired glucose tolerance were examined. The study was a randomized, double-blind, placebo-controlled, crossover study that involved 30 diabetes mellitus patients and 6 impaired glucose patients. Each patient was subjected to 900 ml of hydrogen rich water and 900 ml or pure water for 8 weeks, with a 12-week wash out period. The results revealed that hydrogen rich water increased extra cellular superoxide dismutase and serum adiponectin levels, which is implicated in improving insulin resistance. The results of the study also revealed that hydrogen rich water was able to reduce serum modified LDL levels as well as normalize the glucose tolerance levels in 4 of the 6 total patients. Indicating that hydrogen rich water aids lipid and glucose metabolism. The conclusion made was that hydrogen rich water has benefits in preventing or impeding type 2 diabetes mellitus and insulin resistance, because of its ability to effectively reduce oxidative stress. In spite of these exciting results and conclusions, Kajiyama et al., stated that a much larger clinical study is needed since this study was relatively small and a warning that caution should be used when interpreting the data [32].

There have been quite a few studies involving molecular hydrogen directly as a treatment for the various conditions and secondary complications that comprise or are caused by diabetes mellitus. The studies indicate that hydrogen maybe a novel therapy to diabetic complications and have a possible therapeutic role in diabetes mellitus itself.

### Alzheimer’s disease

Recently new connections concerning the pathogenesis of Alzheimer’s disease and oxidative stress have been made. However, there have only been a few studies directly involving hydrogen therapy in Alzheimer’s disease [33].

A study performed by Li et al., wanted to know if hydrogen rich saline had an effect on inflammation caused by amyloid β and learning and memory. After intracerebral ventricular injection of the amyloid peptide, Aβ1-42, there was an increase in MDA, IL-6, and TNF-α were all observed. After hydrogen rich saline was administered as a treatment, reductions in these parameters were also observed. It is also suggested that the major findings of this study is that hydrogen rich saline was able to improve long-term potentiation, learning, and memory most likely by reducing inflammation and oxidative stress. These assumptions were verified by results of improved response to inflammation and the inhibition of accumulating lipid peroxidation products [34,35].

Evidence that hydrogen rich saline is able to inhibit the activation of c-Jun NH_2_-terminal kinase (JNK) and nuclear factor-κB (NF-κB) has also been shown in the Aβ1-42 Alzheimer’s diseases rat model. The attenuation of JNK and NF-κB is a critical finding since it has been previously shown that amyloid induces apoptosis through oxidative stress resulting from these pathways [35].

### Cerebrovascular diseases

The effects of hydrogen therapy in cebrovascular diseases has shown a lot of promise since there are a myriad of positive results showing molecular hydrogen efficacy in this field of study. The effect of molecular hydrogen therapy has been analyzed in various mediums and in numerous animal models. Cai et al. found that hydrogen gas was able to show anti-apoptotic properties by reducing cell death and limiting the activity of caspase-3 and caspase-12, and thus increasing cell survivability in the neonatal hypoxia ischemia rat model. Cai et al. proposed that hydrogen was able to hinder apoptosis by quelling free radicals that trigger pathways that lead to the activation of caspase-3. Cai et al. also analyzed the short and long term effects of hydrogen rich saline in a neonatal hypoxia ischemia model. The short-term results suggested that hydrogen rich saline was able to reduce the infarct ratio, cell death via apoptosis, oxidative stress, as well as prevent caspase-3 and microglia activity causing an overall long-term neurological improvement after the brain injury [19]. These findings lead to the conclusion that hydrogen therapy may be a possible agent in dealing with hypoxia ischemia and other neonatal brain disorders [19,36].

In addition to antioxidant properties, hydrogen rich saline has also been shown to have anti-inflammatory properties and was able to reduce expression and activity of TNF-α, NF-κB, and IL-6 after transient ischemia and reperfusion in rats [37]. Similar results of a reduction in oxidative stress and inflammation along with an up regulation of Bcl-2 and down regulation of Bax and caspase-3 were seen by Liu et al. in a focal ischemia and reperfusion rat model [38].

Hydrogen rich water has also shown some effectiveness in cerebrovascular injuries by preventing superoxide formation in brain slices of SMP30/SNL knockout mice. Sato et al. speculates that molecular hydrogen may be able to protect against ROS by translocating to the nucleus and affecting gene transcription or by preventing production by acting on the mitochondria itself [39].

Fu et al. found that hydrogen rich water may also be able to play a therapeutic role in impeding the advancement of Parkinson’s Disease. This conclusion was reached after evaluating the results of behavior and pathological examinations and discovering that the application of the hydrogen rich water was able to protect against 6-hydroxydopamine-induced nigrostriatal degeneration by reducing oxidative stress [40]. Related results were also seen by Ito et al. who found that intermittent hydrogen gas exposure seemed to be effective in preventing 6-hydroxydopamine-induced Parkinson’s Disease in rats, although not at the same efficacy as hydrogen rich water [41]. Fujita et al. found similar results in another Parkinson’s Disease rat model. The results conveyed that hydrogen rich water was able to protect against neuronal apoptosis induced by 1-methyl-4pheny-l, 2, 3, 6-tetrahydropyridine by reducing ROS production [42].

A randomized double-blind placebo-controlled trial involving human patients conducted by Yoritaka et al. demonstrated that hydrogen rich water was able to reduce oxidative stress and improve Parkinson’s features. This was indicated by improved Total Unified Parkinson’s Disease Rating Scale scores in a majority of the patients that consumed hydrogen rich water after 48 weeks. Although the results are promising, Yoritaka et al. suggests that longer and larger trials should be conducted in order to fully ascertain the effects of hydrogen rich water in Parkinson’s Disease. To the knowledge of Yoritaka et al. this is the first randomized double-blind, placebo-controlled, parallel-grouptrial involving hydrogen rich water and humans [43]. Domoki et al. are among the first to ever demonstrate the effectiveness of hydrogen supplemented room air ventilation as a therapeutic agent after perinatal asphyxia in a piglet animal model. Treatment with hydrogen supplemented room air ventilation was able to preserve cerbrovascular reactivity and be instrumental in protecting neurons. Which lead to the conclusion that hydrogen supplemented room air can provide early neuroprotection after asphyxia, further studies are needed in order to identify the full capabilities of neuroprotection [22]. Hugyecz et al. also demonstrated how hydrogen supplemented room air ventilation is able to reduce the activity of cyclooxygenase-2 (COX-2) in the hippocampus after transient global cerebral ischemia leading to reduced neuronal damage due to truncated ROS production [20].

Experiments have also shown that hydrogen gas is effective in traumatic brain injuries and has neuroprotective properties as well. Ji et al. demonstrated that hydrogen gas is able to hinder the permeability of the blood brain barrier, reduce brain edema, and decrease neurological dysfuntion by reducing ROS and oxidative stress in rats after the traumatic brain injury [44]. Manaenko et al. also showed that hydrogen gas inhalation is a beneficial agent in an intracerebral hemorrhage mouse model. Hydrogen gas was able to improve neurological functions, decelerate blood brain barrier permeability, as well as decrease the accretion of mast cells causing a decrease in mast-cell specific proteins and production of reactive oxygen and nitrogen species [45].

Although the efficacy results involving molecular hydrogen are positive there are some limitations. Matchett et al. reported that hydrogen gas is ineffective when moderate to severe damage occurred in a neonatal hypoxic rat model. The results indicate that it is possible that severe ischemia damage may have overwhelmed the effects of hydrogen treatment. This indicates that longer exposure to hydrogen, or different concentrations may have a more beneficial effect, or that hydrogen may only be effective in mild cases of brain injury [46].

There are some limitations to hydrogen rich saline as well. It has been shown that hydrogen rich saline may only be able to reduce ROS and damage caused by ROS directly after injury. This limits hydrogen rich saline since clinically most of the damage is caused six to twenty-four hours following injuries [37]. In addition, evidence has shown that the neuroprotective effects of molecular hydrogen may vary by animal model. Although further studies are definitely necessary, based upon the evidence it is reasonable to conclude that molecular hydrogen therapy may be safe and novel therapeutic treatment to various types of brain injuries and cerebrovascular disease [44]. Safe and novel therapy solutions are really needed in the United States, especially since approximately 800,000 American people undergo neurological procedures each year [47].

### Chronic lower respiratory

Molecular hydrogen has been observed as a therapeutic agent in acute and chronic respiratory diseases and in many experiments has shown potential to be a potential therapy. The application of hydrogen has shown different efficacies in relation to the various mediums.

One promising event was the effects of hydrogen rich saline on lung injuries induced by intestinal ischemia and reperfusion observed by Mao et al. In this experiment it was observed that hydrogen rich saline was able to reduce MDA levels after reperfusion when compared to the control group. Also the effects of the hydrogen rich saline treatment could be seen histologically, with the observation of moderate edema inflammatory cell infiltration, and hemorrhage [48].

In addition, Fang et al., demonstrated the effects of hydrogen rich saline by inducing lung injuries by means of an extensive burn rat model. It was found that hydrogen treatment was able to improve the pulmonary oxygenation function in severely burned rats due to its ability to decrease inflammation cascades promoted by TNF-α and IL-1, which resulted in a higher partial pressure to oxygen when compared to the controls [49].

Sun et al., also found similar results and was able to demonstrate that hydrogen rich saline had an effect on hyperoxia lung injuries. The usage of hydrogen rich saline, resulted in reduced levels of pro-inflammatory cytokines and ROS. These reductions lead to reduced lung injury and lung cell apoptosis [50].

Wang et al., who used hydrogen rich saline as treatment for pulmonary hypertension, also reported the familiar outcomes of decreased amounts of pro-inflammatory cytokines. The results of this study also provided evidence of decreased ROS production and increased antioxidant activity. This shows that hydrogen rich saline may in fact be beneficial for pulmonary hypertension in rats [51].

Shi et al., has reported that treatment with hydrogen rich saline may prevent lung cell apoptosis by directly or indirectly affecting caspase-3 in cases of acute pulmonary ischemia and reperfusion. Shi et al. saw decreased amounts of caspase-3 activity when compared to the control groups, indicating that hydrogen may be active in the apoptotic pathway. Since the apoptotic pathway is rather complex and contains a variety of variables further studies should be conducted on the role of hydrogen in the activation of caspase-3 [52].

All these results seem to indicate that hydrogen rich mediums; especially hydrogen rich saline may be an effective treatment for lower respiratory and lung associated diseases by preventing the accumulation of ROS and pro-inflammatory cytokines. Wang et al., concludes that although the results are very promising, the time-course analysis and a thorough mechanism of molecular hydrogen affects on pulmonary hypertension and subsequently all pulmonary or pulmonary associated diseases are necessary to determine whether or not hydrogen rich saline has preventative or even therapeutic effects [51].

### Malignant neoplasms

Malignant neoplasms or cancer is the second cause of death in the United States. The effects of molecular hydrogen in cancerous disease states have been previously documented and it is reported that hydrogen may have therapeutic potential.

It has been shown that hydrogen rich water had an effect on inhibiting tumor growth as a result of its ability to reduce oxidation products. The evidence for molecular hydrogen inhibition of tumor growth was shown by Saitoh et al., Human tongue carcinoma cells HSC-4, cultured with hydrogen showed a decrease in colony numbers by 72% along with the number per colony decreasing by 66%. Human fibrosarcoma cells incubated with the hydrogen rich water also showed similar responses, as a significant decrease in colonies was observed as well [53].

Another observation in cancer research involves hydrogen rich saline. A study found that molecular hydrogen was instrumental in protecting mice from radiation induced thymic lymphomas. The results also showed that hydrogen treatment was able to slow the rate of radiation induced thymic lymphomas as well as prolong the latency period by reducing ROS, which has been found to be a factor of inducing cancers [54].

Although there have been some positive results of hydrogen being a treatment for cancer by reducing ROS more inquiries are needed and required to pass judgment. Especially since ROS production is often employed as a common therapeutic approach to help induce apoptosis in cancer cells and combat cancer. On the other hand, ROS has also been implicated in the induction and conservation of cancer. Thus it is important that more studies be performed in order to fully assess all of the capabilities of hydrogen therapy in cancer [55].

### Cardiovascular

The effects of molecular hydrogen on the cardiovascular system have been fairly well documented when compared to other systems, diseases, and causes of death. Since heart disease is the number one cause of death in America it is imperative to have treatments against the disease. Although the results involving hydrogen and the heart have been positive in animal models, there still has not been any clinical trials or hints of movement toward clinical trials in the near future despite promising results.

Ionizing radiation-induced damage of the heart has been shown to lead to chronic cardiac disease. Irradiation of the heart has been shown to be caused by the hydroxyl radical, intervention to protect the heart from the damage of the hydroxyl radical has been proposed, suggested, and performed by Qian et al., who have investigated the cardioprotective properties of hydrogen by pre-treating mice with hydrogen rich water prior to irradiation. The results were pleasing, 90% of the mice without hydrogen rich water pretreatment died, while 80% of the mice with hydrogen treatment lived after 13 days post-radiation. When focusing on the myocardium, hydrogen pre-treatment proved to have cardioprotective properties by decreasing melanodialdehyde (MDA) and eight-hydroxydeoxyguanosine (8-OHdG) levels as opposed to the non-treatment counterparts, which showed increased levels of those oxidative stress markers [56].

Zhang et al. performed a study to analyze the anti-inflammatory of hydrogen rich saline on rats that received a left anterior descending coronary artery occlusion. The results showed that hydrogen rich saline has anti-inflammatory and cytoprotective properties by decreasing pro-inflammatory cytokines and decreasing myocardial cell damage caused by the adhesion molecule ICAM-1 after ischemia and reperfusion injury [57]. A study performed by Sun et al., demonstrated that hydrogen rich saline was able to be effective against myocardial ischemia and reperfusion injury in rats. The findings of this study were that there were significant decreases in infarct sizes, MDA concentrations and 8-OHdG levels in at risk areas, as well as evidence that hydrogen was able to inhibit the affects of caspase-3, and inhibit apoptosis in cardiomyocytes [13]. Hydrogen gas, at incombustible levels has also exhibited cardioprotective properties. In a study by Hayashida et al., hydrogen gas was shown to reduce myocardial infarct sizes that resulted from ischemia reperfusion injury. The study also suggests and concludes that the use of hydrogen gas may have a clinical application now by using hydrogen gas treatment in conjunction with routine percutaneous coronary intervention procedures [58]. Overall, molecular hydrogen via gas, water or saline has proved to be effective at reducing various types of damage caused by inflammation, radiation, and ischemia and reperfusion. After the reporting the appealing results, Sun et al., concluded in their study that molecular hydrogen specifically “Hydrogen rich saline may, on the basis of our observation, offer a simple, easy to use, safe, and economic novel approach for future cardiac protection” [13].

### The future

Currently there is only one active clinical trial concerning molecular hydrogen as a therapy. The study involves the evaluation of hydrogen administered orally and topically as a therapy for sports-related soft tissue injuries. This study aims to measure and observe changes in serum interleukin 6, plasma viscosity, passive joint flexibility, joint swelling, pain intensity, and serum C-reactive protein. This is currently a Phase 2 clinical trial and hopefully the outcome of this clinical trial will be promising and lead to more clinical trials involving hydrogen rich formulations in the future [24].

It has been reported that many previous antioxidant therapy treatments have had disappointing results in clinical trials [59]. The disappointments and problems that arose resulted in antioxidant therapy being perceived as negative or tainted. This tainted image is thought to be due to a lack of knowledge concerning antioxidants. In order to achieve better results in clinical trials it is proposed that knowledge involving the effects of excess accumulation, the reducing potential, the dosage, the dose duration, and safety of the antioxidant should all be determined [60].

Information about hydrogen as an antioxidant therapy meets all of the proposed requirements to a certain extent. Even though the exact mechanism is unknown, it is also known that molecular hydrogen has the ability to scavenge and reduce hydroxyl radicals, which can lead to a decrease in oxidative stress because of fewer amounts of free radicals and ROS. As for the dosage and dosage duration, a universal standard concentration has not been created, since the effectiveness of hydrogen seems to vary by animal model [44]. However, numerous experiments have been published with promising results from various concentrations of hydrogen administered either via gas, saline, or water. One example can be seen in a recent experiment published by Itoh et al., which made the conclusion that hydrogen is “likely to be instrumental in exerting a protective effect against Parkinson’s disease” [41]. The pilot study that discovered that the scavenging ability of hydrogen significantly improved symptoms of rheumatoid arthritis, also suggests that hydrogen can be used as a therapy in this capacity as well.

The safety of hydrogen has also been explored and documented. The effects of excess accumulation has been reported and speculated to be negligible. It has also been discovered that hydrogen poses no risks for explosion or flammability at concentrations less than 4.7 percent of air.

Hydrogen is often used in the field of diving and has already been safely administered to prevent decompression sickness in divers [2]. Administration of hydrogen rich saline has also been shown to significantly reduce and protect against decompression sickness in rats [61]. This protection is most likely caused by anti-oxidation properties of hydrogen. Which is compatible with the notions that oxidative stress contributes to the development of decompression sickness [62].

## Conclusion

Studies on molecular hydrogen has come a long way from its humble beginnings and has evolved tremendously. There have been significant findings in the research of molecular hydrogen, however progress needs to continue. Molecular hydrogen has been involved in very promising results thus far. In order for hydrogen therapy to be effective in clinical trials and eventually used in medicine, the exact mechanisms of how molecular hydrogen operates, needs to be fully discovered and explored. The missing links of how hydrogen scavenges the hydroxyl radical, and how hydrogen is involved in cell signaling and activation, suppression of pathways, how hydrogen interacts with other antioxidants to promote cytoprotection, and how hydrogen can reduce inflammation are all important. Also the efficacy of specific concentrations in various disease models needs to be determined as well as the optimal forms of administration. The future looks very bright and promising; hopefully hydrogen will be at the forefront of medicine and will make surging strides as simple treatments to the top ailments such as neurodegenerative diseases, cardiovascular diseases, respiratory diseases, and a numerous amount of other diseases.

## Abbreviations

ROS: Reactive oxygen species; MCP: Myelocyte chemoattractant protein; MDA: Melanodialdehyde; Cr: Creatinine; BUN: Blood urea nitrogen; MPO: Myeloperoxidase; 8-OHdG: Eight-Hydroxydeoxyguanosine; NF-κB: Nuclear factor-κB

## Competing interests

The authors declare that they have no competing interests.

## Authors’ contributions

BJD-Role included reviewing manuscripts, review design, manuscript preparation, and manuscript editing. JT-Role included manuscript proof reading. JZ-Role included review design and manuscript proof reading. All authors read and approved the final manuscript.
